# mir-98-5p regulates gluconeogenesis and lipogenesis by targeting PPP1R15B in hepatocytes

**DOI:** 10.1007/s12079-023-00735-0

**Published:** 2023-03-14

**Authors:** Rukshar Khan, Amit Kumar Verma, Malabika Datta

**Affiliations:** 1 CSIR-Institute of Genomics and Integrative Biology, Mall Road, Delhi, 110007 India; 2 Academy of Scientific and Innovative Research, CSIR-HRDC, Kamala Nehru Nagar, Ghaziabad, Uttar Pradesh 201002 India; 3 Jamia Millia Islamia, Jamia Nagar, Okhla, Delhi, New Delhi 110025 India

**Keywords:** miRNA, Diabetes, Liver, PPP1R15B, Gluconeogenesis, Steatosis, p-eiF2α, Circulation, NAFLD, Hepatic glucose output, CREB

## Abstract

Several reports suggest that circulatory miRNAs are deregulated in diverse diseases and used as markers for disease diagnosis and prognosis. Here we show that miR-98-5p, that is down-regulated in the circulation during diabetes, regulates hepatic gluconeogenesis and lipogenesis by targeting PPP1R15B. miR-98-5p overexpression significantly decreased the transcript and protein levels of PPP1R15B in hepatic HepG2 cells and increased p-eIF2α expression and these were prevented in the presence of its inhibitor. Two major hepatic hallmarks during diabetes i.e. hepatic lipid accumulation and glucose output were explored towards physiological relevance. As compared to scramble, overexpression of miR-98-5p decreased the transcript levels of both gluconeogenic and lipogenic genes together with a significant reduction in hepatic glucose production and fat accumulation in HepG2 cells. Using PASTAA to detect common transcription factors regulating these altered genes, CREB emerged as the most significantly enriched transcription factor. While miR-98-5p overexpression did not change the transcript levels of CREB, there was a significant change in its protein levels. While similar effects on gluconeogenic and lipogenic gene expression were detected using the PPP1R15B siRNA, the opposite was observed in the presence of miR-98-5p inhibitor alone. All these suggest that by targeting PPP1R15B, miR-98-5p regulates hepatic steatosis and glucose output; deregulation of which are characteristic hepatic features during diabetes. Therapeutic intervention of the miR-98/PPP1R15B axis might offer a potential strategy to target aberrant hepatic metabolism during diabetes.

**Graphical Abstract:**

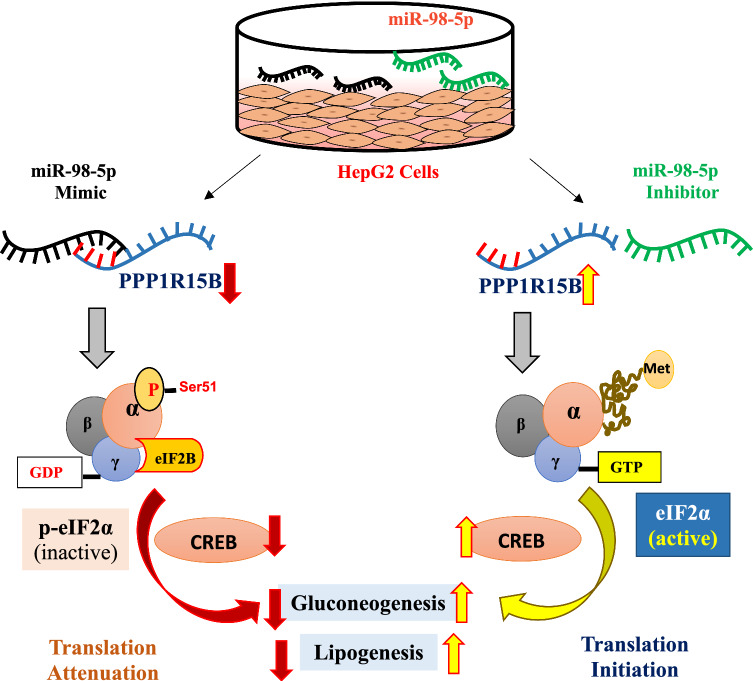

## Introduction

Diabetes mellitus is a chronic, metabolic state attributable to a combination of decreased insulin production by pancreatic-β cells and insulin resistance in insulin-target tissues (Hurtado and Vella 2019, Galicia-Garcia, Benito-Vicente et al. 2020). The liver, skeletal and cardiac muscle, fat, and brain are among the tissues that contribute to the maintenance of normal blood glucose levels. Liver is the central metabolic tissue crucial in maintaining glucose and lipid homeostasis that is affected by impaired insulin action (Loria et al. 2013; Rui 2014). It accounts for approximately 80% of endogenous glucose production and during starved states, it serves as the primary source of glucose via glycogen mobilisation and hepatic gluconeogenesis. However, abnormal activation of hepatic gluconeogenesis and/or abrogation of insulin mediated inhibition of gluconeogenesis results in increased hepatic glucose production and elevated hepatic de novo lipogenesis. Such abrogation of insulin action or insulin resistance is a key component in the development of Non-Alcoholic Fatty Liver Disease (NAFLD) (Ota et al. 2007; Gaggini et al. 2013, Kitade et al. 2017).

In the past few years, microRNAs (miRNAs) have been identified as regulators of gene expression that control biological processes by fine-tuning the levels of target mRNAs. Altered signatures of miRNAs, especially in the circulation are identified as promising biomarkers in various diseases like obesity, cancer, neurodegenerative diseases, atherosclerosis and other metabolic disorders (Mori et al. 2019). Circulatory miRNAs originate from tissues throughout the body(Sapp and Hagberg 2019) and are released by cells into the blood or other biofluids (Valadi et al. 2007) and remain bound to proteins or are encapsulated in extracellular vesicles (EVs). Such miRNAs on EVs may act as inter-organ signalling mediators without being degraded by extracellular/intracellular RNases during their transport (Yáñez-Mó et al. 2015, Huang, Huang et al. 2020). They are secreted and taken up by recipient cells where they are biologically active (Jansen et al. 2013) and exert their respective cellular functions. Increased circulating levels of miR-15a lead to the prediabetic state and impairs glucose tolerance (Kamalden et al. 2017). Circulatory miR-20b levels are increased in T2D patients and this has been shown to correlate with compromised insulin signaling and decreased muscle cells’ capacity to produce glycogen (Katayama et al. 2019). Also, miR-103 levels are altered during insulin resistance and NAFLD and it is identified as a molecular connection between these states (Xu et al. 2015). A previous report from our laboratory had shown that decreased circulatory levels of miR-98-5p in T2D subjects affect apoptosis and proliferation of keratinocytes within the skin (Khan et al. 2020). These reports suggest that a correlation exists between circulatory miRNAs and their action within tissues that determines the fine-tuning of cellular pathways.

In this study, we sought to explore the effects of miR-98-5p on hepatic gluconeogenesis and lipogenesis, and correlate the consequences of the altered circulatory levels of this miRNA to aberrant hepatic physiology during diabetes.

## Materials and methods

### miRNA target prediction

Predicted targets of miR-98-5p were extracted from miRDB (www.mirdb.org), TargetScan (www.targetscan.org) and PicTar (www.pictar.mdc-berlin.de) and a consensus list of targets common to all the three tools was considered to prioritize the genes taken for validation.

## Cell culture and transfection

Experiments were done in the human hepatic cell line (HepG2) obtained from the National Centre for Cell Science, Pune, India and maintained in Dulbecco’s modified Eagle’s medium (Sigma, St. Louis, MO, USA) supplemented with 10% fetal bovine serum and antibiotic/antimycotic (GIBCO Laboratories, Grand Island, NY, USA) in an atmosphere of 5% CO_2_ at 37ºC. HepG2 cells were plated in six-well plates and at 40% confluence, they were transfected with either the scramble or miR-98-5p mimic (25 and 50 nM) with or without its inhibitor (75 nM) (Dharmacon, Lafayette, CO, USA) or miR-98-5p inhibitor alone using Lipofectamine 2000 (Invitrogen, CA, USA) and Opti-MEM (Invitrogen, CA, USA) according to the manufacturer’s instructions. Cells were incubated for 48 h and on termination of incubation, total RNA was isolated or cells were lysed for proteins as described below. Also, cells were transfected with either the scramble or PPP1R15B siRNA (25 and 50 nM) for 48 h and evaluated for levels of gluconeogenic and lipogenic genes.

### Quantitative reverse transcriptase (RT)-PCR

On termination of incubation, total RNA was isolated using TRIzol (Ambion, Life Technologies, Germany) and quantified using Infinite 200 PRO plate reader (Tecan, MAENNEDORF, Switzerland). The 260/280 ratio was between 1.9 and 2.0. cDNA was synthesized by reverse transcribing 1 µg of RNA using random hexamers and the cDNA was subjected to Real-Time PCR (Applied Biosystems, CA, USA) using SYBR-Green PCR Master Mix (Applied Biosystems, CA, USA) and transcript levels of PPP1R15B, CREB, PCK1, G6PC, FBP, PC, ACC, ACLY, FASN, ADRP, HMGCS, and HMGCR were quantified using specific primers (Table 1). 18S rRNA was used as the endogenous control. Relative gene expression was calculated using the 2 − ΔΔCt method.

**Table 1:** Primers for qRT-PCR and cloning

Gene name	Forward primer (5′-3′)	Reverse primer (5′-3′)
PPP1R15B	tgaggattgggatgaggaag	tctggcagcagtctgaattg
18 S rRNA	agaaacggctaccacatcca	agtgtgacgttgacatccgta
PPP1R15B 3’UTR	ccgctcgaggagagaagtaatggcaaggcc	ataagaatgcggccgcgtgtgtgcctgtaatcccag
Mutant 3’UTR	gaatttttcatcagaagtgcttacagggttacgcaatcagtttacaatctacctggtcattattttat	ataaaataatgaccaggtagattgtaaactgattgcgtaaccctgtaagcacttctgatgaaaaattc
PCK1	ggttcccagggtgcatgaaa	cacgtagggtgaatccgtcag
G6PC	tctacgtcctcttccccatc	tcagtatccaaaacccaccag
FBP	tttctgtaccccgctaacaag	tgaatgtctgtgggaatgacg
PC	gactctgtgaaactcgctaaac	ctctgtgaccgtgtgctc
ADRP	ccctcctgtccaacatccaa	ggaggctgtcagacacttct
FASN	ggaggagtgtaaacagcgct	ttggcaaacacaccctcctt
HMGCR	agcctgggccagagaagata	ggcacagttctagggccatt
HMGCS	gactgtcctttcgtggctca	gcagtctccaggtctgtcac
ACC	tgtccaaggggccatgttat	ccccaagaaaagcagtgacc
ACLY	cactctggatggggtcaagt	ccttctcgggtggcatagat
CREB	aaacagagtggcagtgcttg	agccagtccattttccacct

### Western blotting

Transfected cells were lysed in RIPA lysis buffer (Sigma, St. Louis, MO, USA) containing protease inhibitors (Calbiochem, Darmstadt, Germany) and 30–50 µg of protein samples were resolved on SDS-PAGE, transferred to nitrocellulose membranes (Merck Millipore Limited, Ireland) and probed with anti-PPP1R15B (1:1000; Abcam, Cambridge, UK), anti-eIF2α (1:1000; CST), and anti-p-eIF2α (1:1000; CST), anti-CREB (1:1000; Santa Cruz) antibodies. Subsequent detection was done with HRP-linked appropriate secondary antibodies (Genei, Bangalore, India), followed by detection with the ECL Western blotting kit (GBiosciences, MO, USA). HSC70, vinculin or β-actin was used as loading control. Densitometric analyses of the blots were done using the AlphaEaseFC Imaging Analysis software (Alpha Innotech Corporation, San Leandro, CA). A rectangular box was drawn surrounding each band and pixel intensity of each band was normalized to the background intensity.

### Cloning, mutagenesis, and luciferase assays

Although previously validated in keratinocytes (Khan et al. 2020), here we attempted to validate the miR-98-5p-PPP1R15B interaction in HepG2 cells. Luciferase reporter constructs harboring the 3′ untranslated region (UTR) of the human PPP1R15B gene spanning the binding sites for miR-98-5p were generated downstream of Renilla luciferase in a psiCheck2 vector (Promega Corporation, Madison, WI) using primers as in Table 1. Substitution mutations (UACC to GCAA) were generated using a site-directed mutagenesis kit (Agilent Technologies, Santa Clara, CA, USA) using mutation-specific primers (Table 1). HepG2 cells were cultured in 12-well plates and at 70–80% confluence, cells were transfected with either the wild-type or mutated plasmid (100 ng) together with either the miR-98-5p mimic and/or the miR-98-5p hairpin inhibitor or the miR-98-5p inhibitor alone using Lipofectamine™ 2000 (Invitrogen, CA, USA) and Opti-MEM (Invitrogen, CA, USA). Cells were lysed after 48 h of transfection and a dual luciferase assay was performed (Promega Dual Luciferase Assay Kit, Madison, USA) according to the manufacturer’s instructions and luminescence was measured on an Infinite M200 Pro Multimode Reader (TECAN, Männedorf, Switzerland). Renilla luciferase values were normalized to those of firefly luciferase.

### Bodipy staining

HepG2 cells plated onto sterilized cover-slips placed on six-well plates were transfected with either the scramble or with the miR-98-5p mimic (50 nM) either alone or together with its inhibitor (75nM) for 48 h as described above. On termination of incubation, cells were washed with PBS to remove cell debris and fixed with 4% formaldehyde (Merck, Germany) for 1 h. Staining with Bodipy (1:10,000) was carried out for 15 min. Cells were then washed thoroughly, counterstained with DAPI (Invitrogen, CA, USA) and viewed in a fluorescence microscope (Tokyo, Japan) at 493/503 nm. Similar lipid quantification was performed in cells transfected with either the scramble or the PPP1R15B siRNA (50 nM, 48 h). Quantification of the green fluorescence was done using the online Image J software (https://imagej.nih.gov/ij/).

### Glucose production assay

HepG2 cells were transfected with either the scramble or with miR-98-5p mimic (25–50 nM) with or without its inhibitor (75 nM) for 48 h as described above. Also, cells were transfected with either the scramble or PPP1R15B siRNA (50 nM, 48 h). Prior to completion of incubation, cells were serum starved for 2 h and then incubated in glucose production media (DMEM-glucose free, 20 mM sodium lactate, 2 mM pyruvate, and 0.5% BSA) for 4 h. Glucose concentrations in the media were measured using Glucose Colorimetric/Fluorometric Assay Kit (Sigma, St. Louis, MO, USA) and normalized to the total protein content of cells.

### Analysis of transcription factors (TFs)

The TFs associated with a set of co-regulated gluconeogenic and lipogenic genes were predicted using the online PASTAA tool (http://trap.molgen.mpg.de) (Roider et al. 2009). This tool utilizes the prediction of binding affinities of a TF to promoters of a set of genes that determines coherence in their regulation and expression. A p-value score of > 0.01 was used to find the top ranked TFs.

### Statistical analysis

Statistical significance for all the experiments was calculated using Student’s t-test, and values are presented as means ± SEM; *P* < 0.05 was considered to be statistically significant.

## Results

### PPP1R15B is a target of miR-98-5p

A previous study from our laboratory showed that circulatory levels of miR-98-5p are significantly down-regulated in diabetic individuals(Khan et al. 2020) and by targeting PPP1R15B, this miRNA was shown to affect apoptosis and proliferation within keratinocytes. miRNAs are known to exhibit tissue specific gene targeting(Ludwig et al. 2016); we, therefore, sought to explore this interaction in hepatic HepG2 cells since the liver is richly supplied with blood vessels and therefore prone to be affected by altered circulatory miRNAs and consequently study the effects of this axis in hepatic metabolism.

**Fig. 1:**
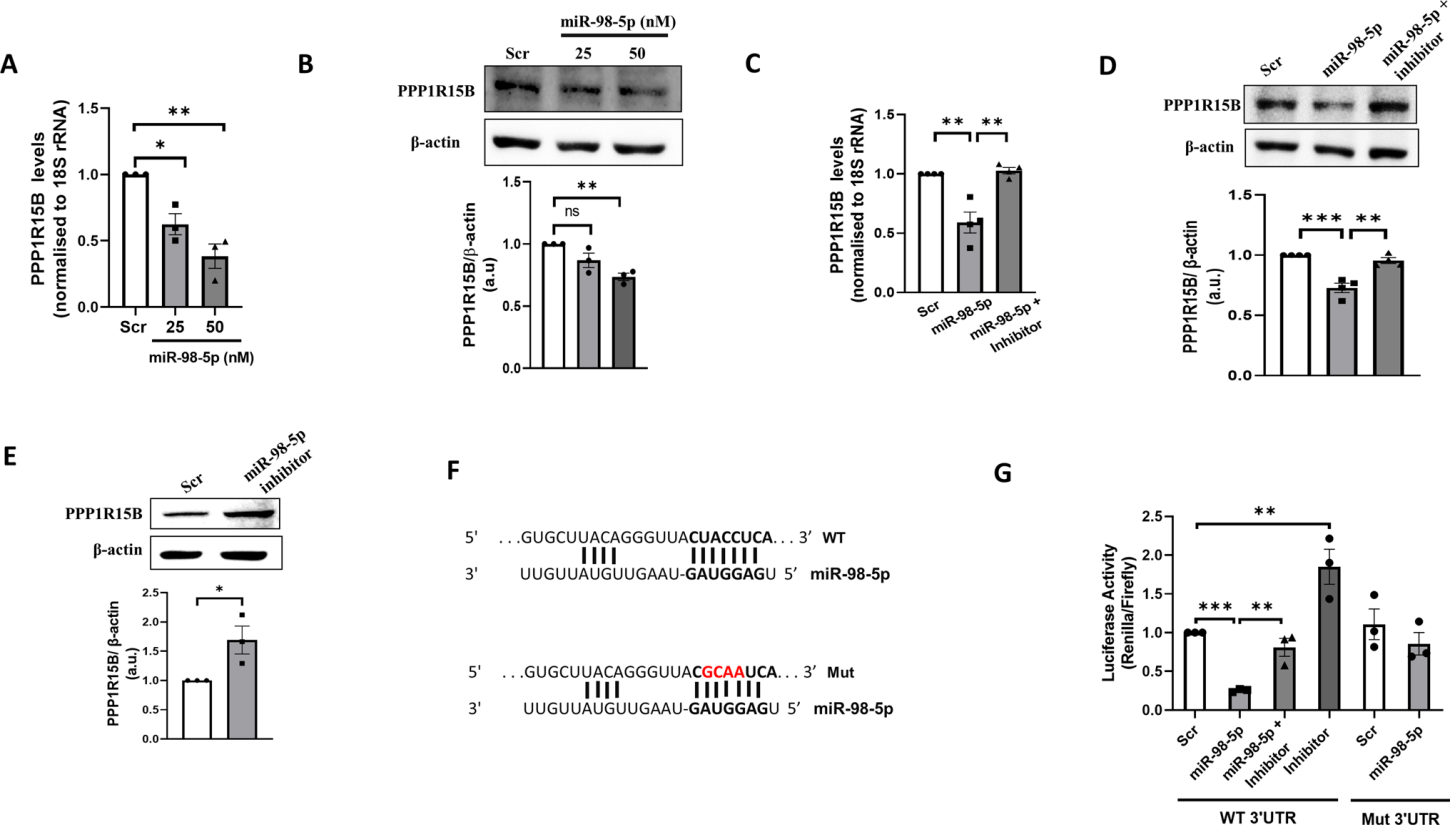
miR-98-5p downregulates PPP1R15B levels in HepG2 cells. **(A)** HepG2 cells were transfected with either the scramble or miR-98-5p mimic (25 and 50nM) and after 48 h, total RNA was isolated, reverse transcribed and subjected to qRT-PCR using primers for PPP1R15B. 18 S rRNA was used as the loading control. **(B)** HepG2 cells transfected with either the scramble (Scr) or miR-98-5p mimic (25 and 50nM) and were lysed after 48 h and 50ug nuclear lysate was subjected to western blot analysis using anti-PPP1R15B antibody. β-actin was used as the loading control. **(C)** Cells were transfected with either the scramble or miR-98-5p mimic and/or inhibitor (75nM) and after 48 h, total RNA was isolated and the transcript levels of PPP1R15B were assessed by qRT-PCR. 18 S rRNA was used as the loading control. **(D)** Cells were transfected as in “C” and on termination of incubation (48 h), the protein levels of PPP1R15B were detected by Western Blot analysis. **(E)** Cells were transfected with either the scramble or miR-98-5p inhibitor (75nM) and after 48 h, the protein levels of PPP1R15B were detected by Western Blot analysis as in “B”. β-actin was used as the loading control. Densitometric analyses are given along with the respective blots. **(F)** The miR-98-5p binding site on the PPP1R15B 3′UTR and the mutation incorporated in the binding site (red) is shown. **(G)** HepG2 cells were plated in 12-well plates and transfected with the wild-type (WT) or the mutated (mut) PPP1R15B ‘UTR’ together with the miR-98-5p mimic (50 nM) and/or its inhibitor (75 nM). Control cells were transfected with the scramble sequence. After 48 h, cells were lysed and luciferase activity was measured as described in the Materials and methods section. Renilla luciferase plasmid was used as the transfection control and firefly luciferase values were normalised to its values. All experiments were done in triplicates and values are presented as means ± SEM. ***p < 0.001, **p < 0.01 and *p < 0.05

From a consensus list of miR-98-5p target genes (293 genes) extracted using three miRNA prediction online tools (miRDB, TargetScan and PicTar), PPP1R15B was prioritised since it is implicated in diabetes and is identified as an underlying monogenic cause of maturity-onset diabetes of the young (MODY)(Abdulkarim et al. 2015, Kernohan et al. 2015). PPP1R15B harbors a binding site for miR-98-5p on its 3’UTR spanning across nucleotides 1433–1440. Towards validation, HepG2 cells were transfected with miR-98-5p mimic (25 and 50 nM). After 48 h, cells were lysed and the levels of PPP1R15B were evaluated. There was a dose dependent decrease in the protein and transcript levels of PPP1R15B with increasing doses of miR-98-5p (Fig. 1A, B). This decrease in PPP1R15B levels was significantly prevented in the presence of the miR-98-5p inhibitor (75nM) (Fig. 1C, D). These suggest that miR-98-5p targets PPP1R15B in HepG2 cells. Also, inhibiting endogenous levels of miR-98-5p using its specific inhibitor significantly increased the endogenous PPP1R15B protein levels as shown in Fig. 1E. To further substantiate this, a PPP1R15B 3’UTR luciferase reporter construct (wild-type) and a plasmid with mutations within the miR-98-5p binding site were constructed (Fig. 1F). HepG2 cells were co-transfected with either the wild-type or mutated 3’UTR reporter plasmids along with miR-98-5p mimic and/or miR-98 inhibitor. As compared to cells transfected with the scramble, luciferase activity of the wild-type plasmid 3’UTR in cells transfected with miR-98-5p was significantly decreased (Fig. 1G) suggesting that miR-98-5p binds to the PPP1R15B 3′ UTR and inhibits luciferase expression. Also, this decrease was prevented in the presence of the miR-98-5p inhibitor and by mutation in the miRNA binding site.

Taken together these results suggest that as in keratinocytes(Khan et al. 2020), this microRNA regulates PPP1R15B levels by binding to its 3′ UTR and decreases both, its mRNA and protein levels in hepatic HepG2 cells.

### mir-98-5p overexpression increases p-eIF2α levels in HepG2 cells

**Fig. 2:**
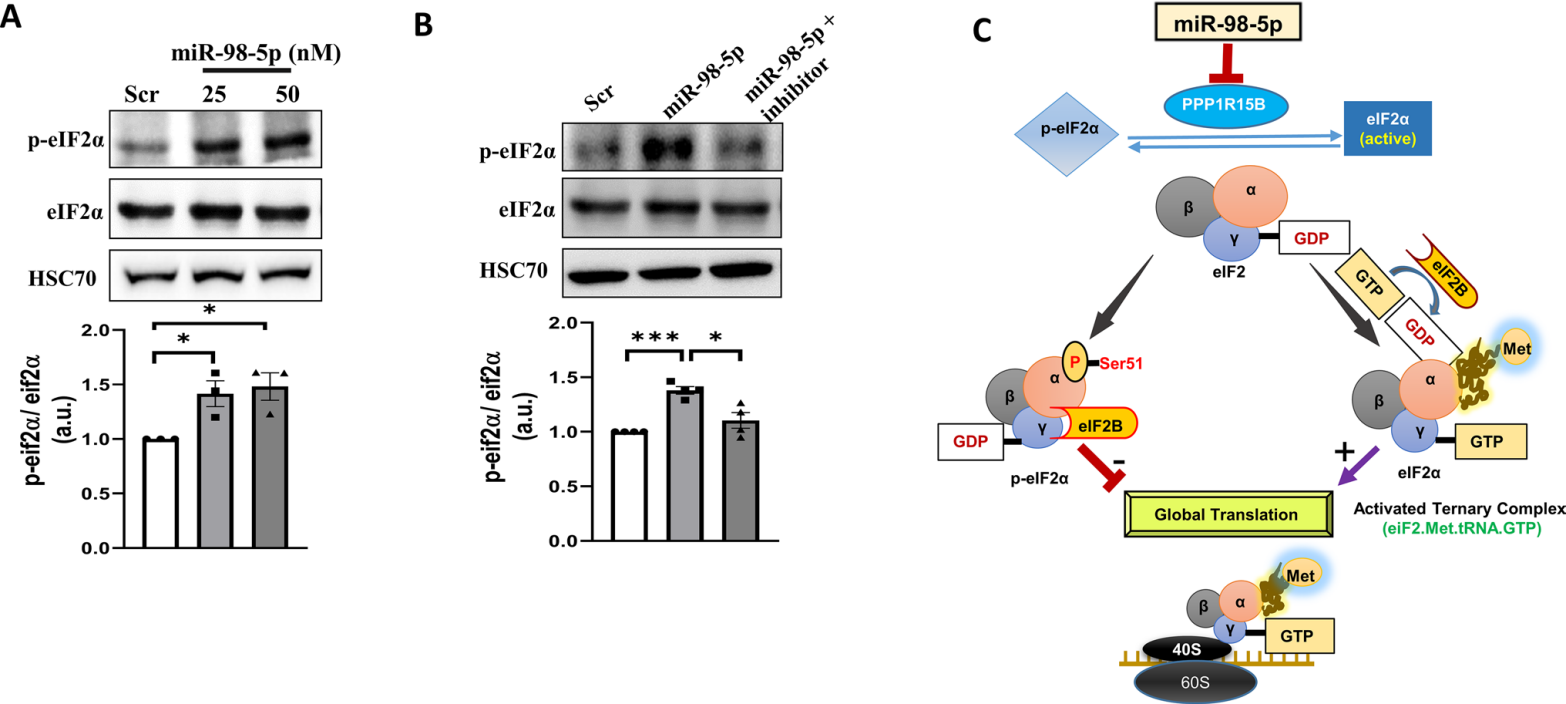
miR-98-5p induces p-eIF2α in HepG2 cells. **(A)** HepG2 cells transfected with either the scramble (Scr) or miR-98-5p mimic (25 and 50nM) and were lysed after 48 h and 50ug lysate was subjected to western blot analysis using anti-eIF2α and anti-p-eIF2α antibodies. HSC70 was used as the loading control. **(B)** Cells were transfected with either the scramble or miR-98-5p mimic and/or inhibitor and after 48 h, cells were lysed and lysates (50ug) were subjected to western blot analysis as mentioned in “A”. HSC70 was used as the loading control. **(C)** Diagrammatical representation of the possible cellular consequences of miR-98-5p overexpression. miR-98-5p targets PPP1R15B and this increases p-eIF2α levels. eIF2α is a component of the trimeric eIF2 protein that upon GTP binding, interacts with the ribosomal complex to initiate translation. However, phosphorylation of the eIF2α subunit at Ser51 prevents GTP exchange and the binding of the ribosomal initiator complex to the GDP-bound p-eIF2 complex is impaired and this stalls translational initiation and halts protein synthesis. Densitometric analysis of the blot is given below. Experiments were performed in triplicate and values are presented as means ± SEM. ***P < 0.001, **P < 0.01 and *P < 0.05

PPP1R15B is a protein phosphatase and it functions to catalyze the dephosphorylation of eIF2α and maintains a balance between p-eIF2α and eIF2α within the cell. Such a balance is very crucial in maintaining the cellular homeostasis during normal and stressed states. HepG2 cells were transfected with miR-98-5p mimic at doses of 25 and 50nM, lysed and the levels of p-eIF2α and total eIF2α were evaluated. As shown in Fig. 2A, there was a dose dependent increase in the levels of p-eIF2α in the presence of miR-98-5p, although the total levels of eIF2α remained unchanged. This increase was prevented in the presence of miR-98-5p inhibitor (Fig. 2B), suggesting that as miR-98-5p targets PPP1R15B and decreases its levels, there occurs a concomitant increase in the levels of p-eIF2α. This flow of regulatory events by miR-98-5p is shown in Fig. 2C where overexpression of miR-98-5p targets PPP1R15B that induces p-eIF2α accumulation, possibly resulting in translational attenuation. eIF2α is an eukaryotic initiation factor of translation and functions by forming a ternary complex with GTP and initiator tRNA during protein synthesis. This complex mediates ribosomal recognition of the start codon during the scanning process by the ribosomal complex to initiate protein translation. However, phosphorylation of eIF2α withdraws this regulatory role of eIF2α on protein translation and consequently protein synthesis is stalled.

### mir-98-5p overexpression inhibits gluconeogenesis in HepG2 cells

**Fig. 3:**
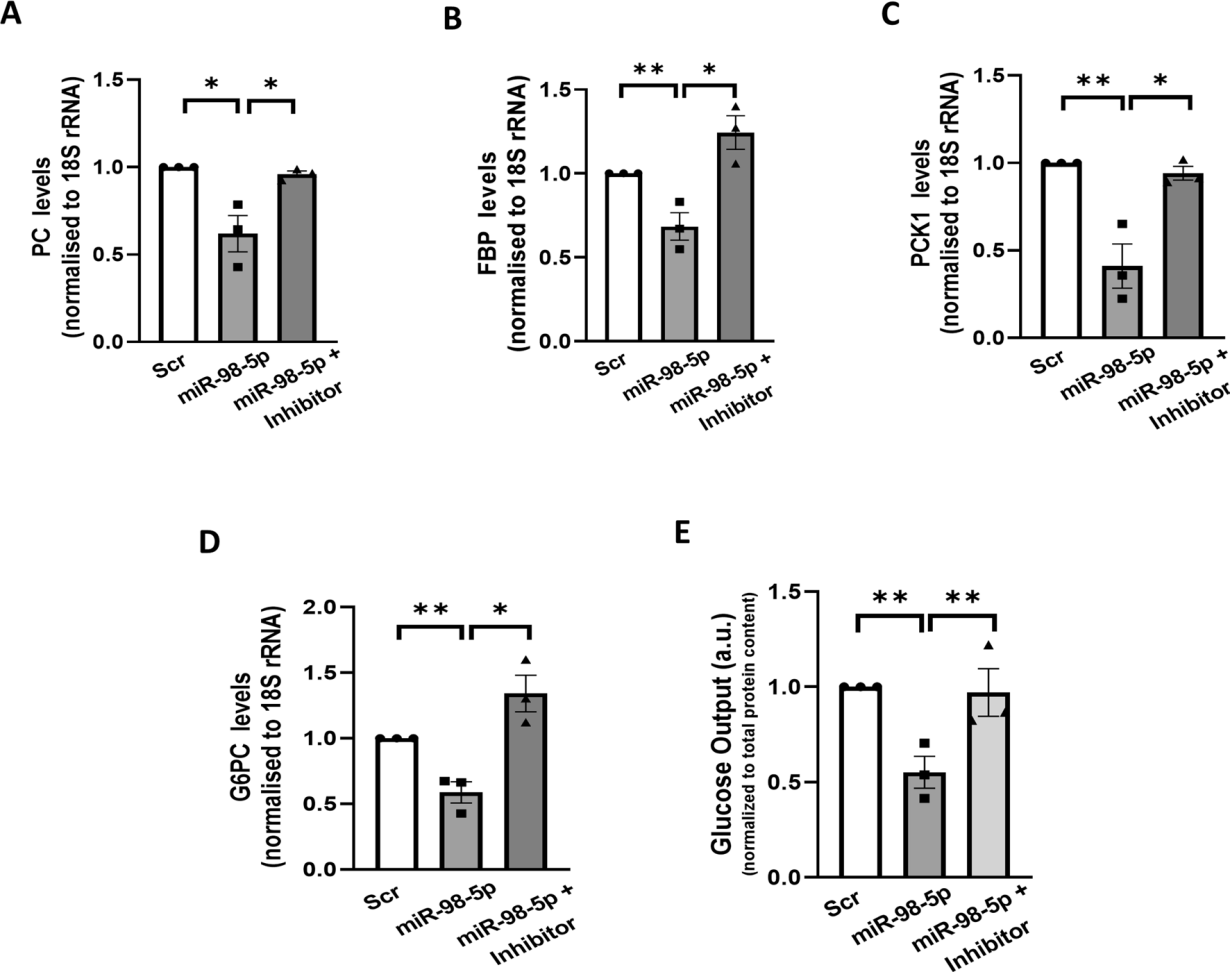
miR-98-5p reduces gluconeogenesis in HepG2 cells. Cells were transfected with either the scramble or miR-98-5p mimic and/or inhibitor and after 48 h, total RNA was isolated, reverse transcribed and subjected to qRT-PCR using gene-specific primers of PC **(A)**, FBP **(B)**, PCK1 **(C)**, and G6PC **(D)**. 18 S rRNA was used as the loading control. **(E)** HepG2 cells were transfected as in “A” and on termination of incubation, glucose output in the media was measured as described in the “Methods” section. Total protein content from whole cell lysates was used for normalization. Experiments were performed in triplicate and values are presented as means ± SEM. **P < 0.01 and *P < 0.05

Results from the above suggest that increased levels of miR-98-5p promote accumulation of p-eIF2α within the cells by targeting PPP1R15B. To determine the consequent effects of miR-98-5p-PPP1R15B interaction mediated p-eIF2α accumulation, we assessed the impact on two evident aberrant hepatic hallmarks during diabetes: gluconeogenesis and lipogenesis. HepG2 cells were transfected either with the scramble or with miR-98-5p mimic alone or together with its inhibitor for 48 h and upon completion of incubation, transcript levels of the gluconeogenic genes (PC, FBP PCK1 and G6PC) were evaluated by qRT-PCR. As shown in Fig. 3A-D, miR-98-5p decreased the mRNA levels of all gluconeogenic genes and such effects of miR-98-5p on gluconeogenic genes’ expression were prevented in the presence of the miR-98-5p inhibitor. Since we observed changes in the status of gluconeogenic genes, we investigated the effects on hepatic glucose production (HGP). The liver maintains glucose levels by a complex balance between endogenous glucose synthesis and glucose absorption through coordination of both gluconeogenesis and glycogenolysis. HepG2 cells were cultured as mentioned in “Materials and Methods” section and transfected with the miR-98-5p mimic alone or together with its inhibitor. As shown in Fig. 3E, as compared to cells transfected with the scramble, hepatic glucose production was significantly ameliorated in the presence of miR-98-5p. This decrease in glucose production was abrogated in the presence of the miR-98-5p inhibitor. These suggest a significant role of this miRNA in altered glucose metabolism in HepG2 cells.

### mir-98-5p overexpression inhibits Lipogenesis in HepG2 cells

**Fig. 4:**
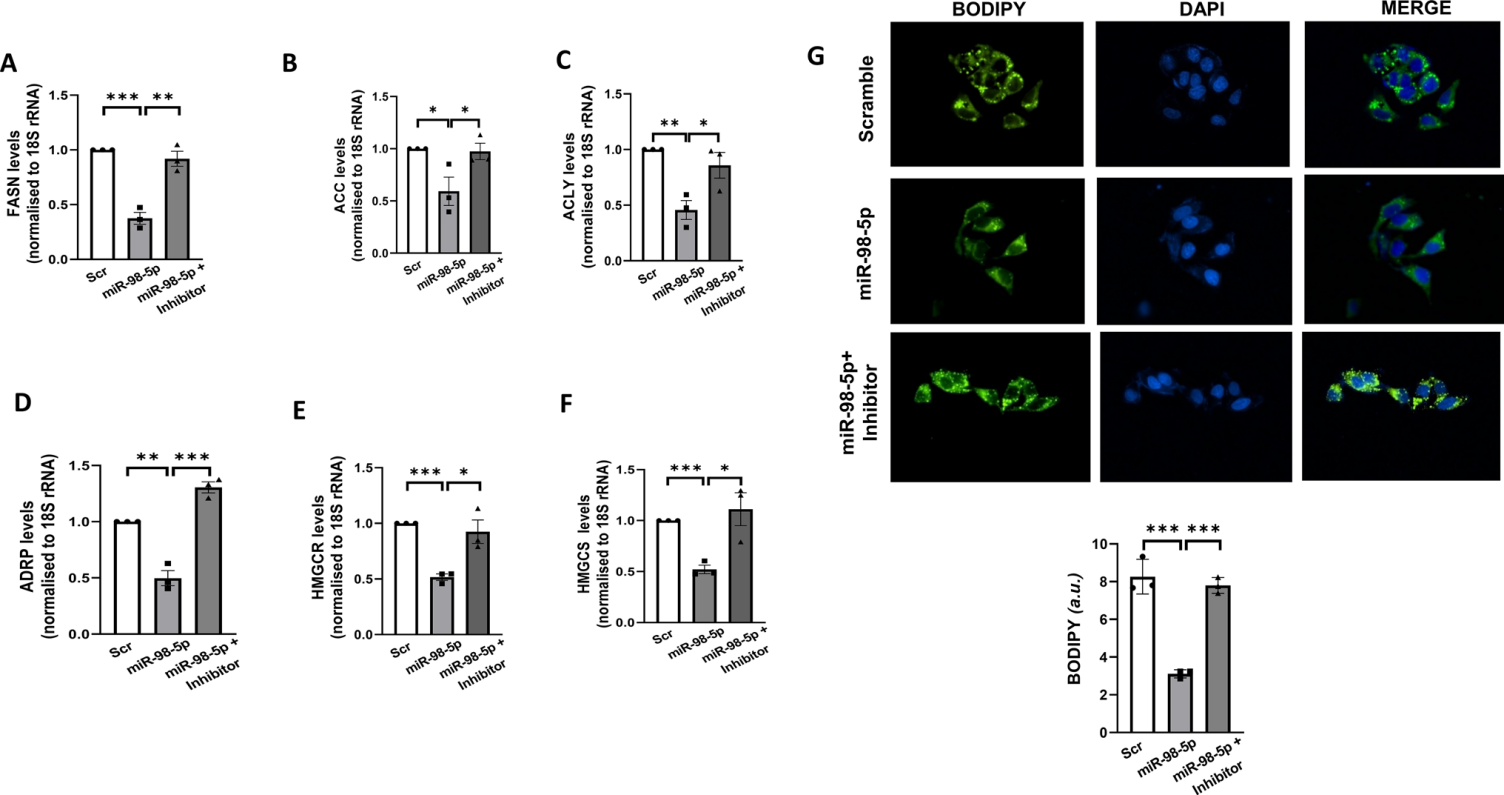
miR-98-5p impedes lipogenesis in HepG2 cells. Cells were transfected with either the scramble or miR-98-5p mimic and/or inhibitor and after 48 h, total RNA was isolated, reverse transcribed and subjected to qRT-PCR using gene-specific primers of FASN **(A)**, ACC **(B)**, ACLY **(C)**, ADRP **(D)**, HMGCR **(E)** and HMGCS **(F)**. 18 S rRNA was used as the loading control. (E) HepG2 cells were grown on cover-slips placed on 24-well plates transfected as in “A”. After 48 h, cells were fixed with 4% formaldehyde and stained with Bodipy for 15 min. Nuclei were stained with DAPI. After thorough washing, cells were visualised in a fluorescent microscope. Quantification of the green fluorescence was done using the ImageJ software and is represented in the lower panel. Experiments were performed in triplicate and values are presented as means ± SEM. ***P < 0.001, **P < 0.01 and *P < 0.05 as compared to scramble transfected cells (Scr)

To determine the effects of miR-98-5p-PPP1R15B interaction, we assessed the impact on another major hepatic hallmark during diabetes i.e. lipogenesis as mentioned previously. HepG2 cells were transfected either with the scramble or with miR-98-5p mimic alone or together with its inhibitor for 48 h and upon completion of incubation, transcript levels of the lipogenic genes (FASN, ACC, ACLY, ADRP, HMGCR, and HMGCS) were evaluated. As shown in Fig. 4A-F, miR-98-5p decreased the transcript levels of lipogenic genes. Also, such effects were prevented significantly in the presence of the miR-98-5p inhibitor suggesting a possible role of this miRNA in altered hepatic lipid metabolism. Since we observed changes in the status of lipogenic genes, we probed the effects on hepatic lipid accumulation. HepG2 cells were cultured as mentioned in “Materials and Methods” section and transfected with the miR-98-5p mimic alone or together with its inhibitor. As shown in Fig. 4G, as compared to cells transfected with the scramble, lipid accumulation (as visualised by Bodipy staining) was significantly decreased in the presence of miR-98-5p at 48 h post transfection. This decrease in cellular lipid content was abrogated in the presence of the miR-98-5p inhibitor, suggesting that miR-98-5p targets PPP1R15B and prevents lipid accumulation in HepG2 cells.

### PPP1R15B inhibition prompts p-eIF2α accumulation and decreases gluconeogenesis and lipogenesis in HepG2 cells

**Fig. 5:**
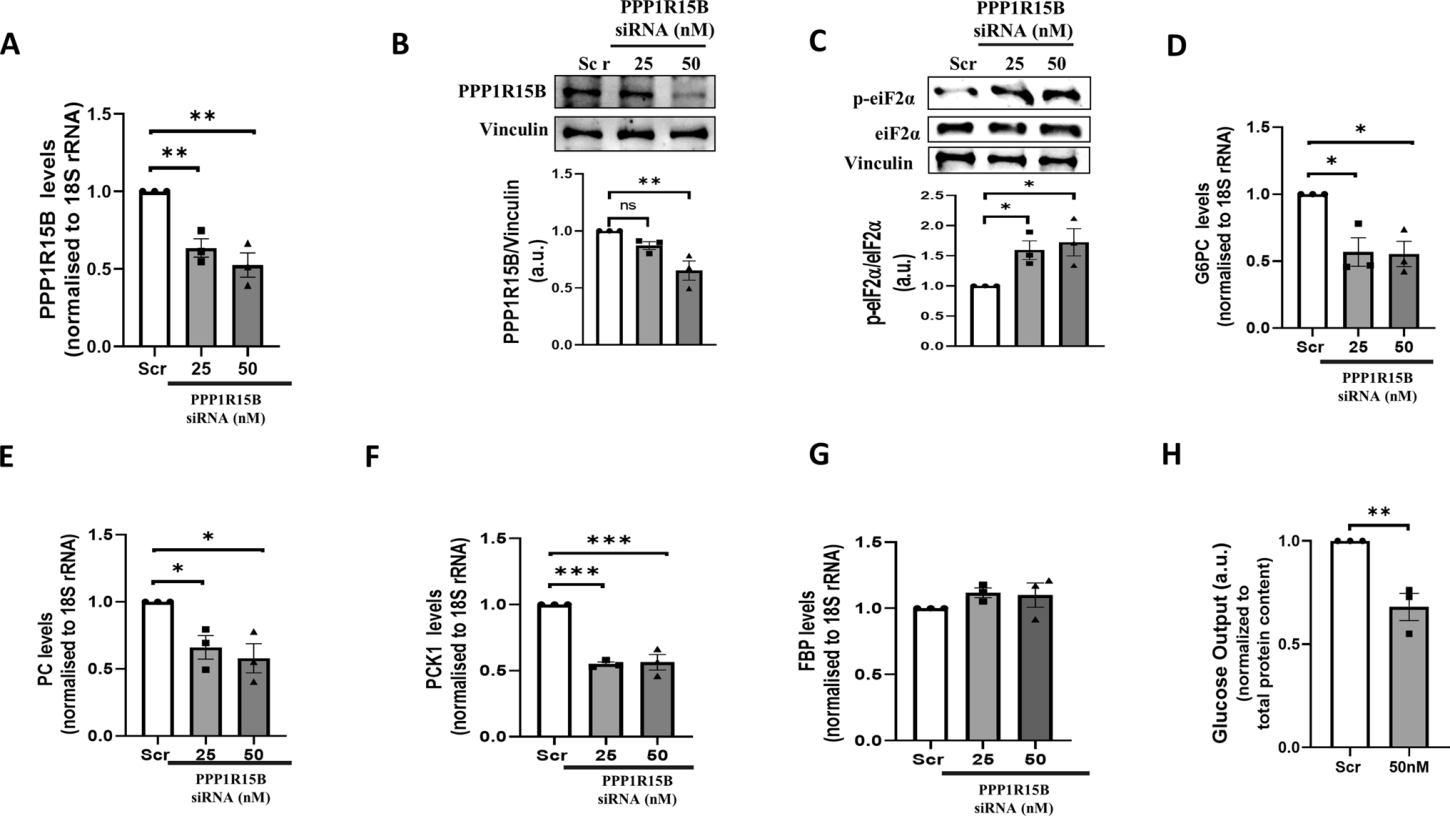
PPP1R15B inhibition induces p-eIF2α accumulation and decreases gluconeogenesis and hepatic glucose output in HepG2 cells. **(A)** HepG2 cells were transfected with either the scramble (Scr) or PPP1R15B siRNA (25 and 50nM) and after 48 h, the levels of total RNA were isolated, reverse transcribed and PPP1R15B transcript levels were quantified by qRT-PCR using specific primers. 18 S rRNA was used as the loading control. HepG2 cells transfected as in “A” were lysed and lysates (50ug) were subjected to western blot analysis using anti-PPP1R15B **(B)** and p-eIF2α/eIF2α **(C)** antibodies. Vinculin was used as the loading control. Cells were transfected as described in “A” and on termination of incubation, total RNA was isolated, reverse transcribed and subjected to qRT-PCR to assess the transcript levels of G6PC **(D)**, PC **(E)**, PCK1 **(F)** and FBP **(G)**. 18 S rRNA was used as the loading control. **(H)** Cells were transfected with either the scramble or PPP1R15B siRNA (50nM) and on termination of incubation (48 h), glucose output in the media was measured as described in the “Methods” section. Total protein content from whole cell lysates was used for normalization. Experiments were performed in triplicate and values are presented as means ± SEM. ***P < 0.001, **P < 0.01 and *P < 0.05

**Fig. 6:**
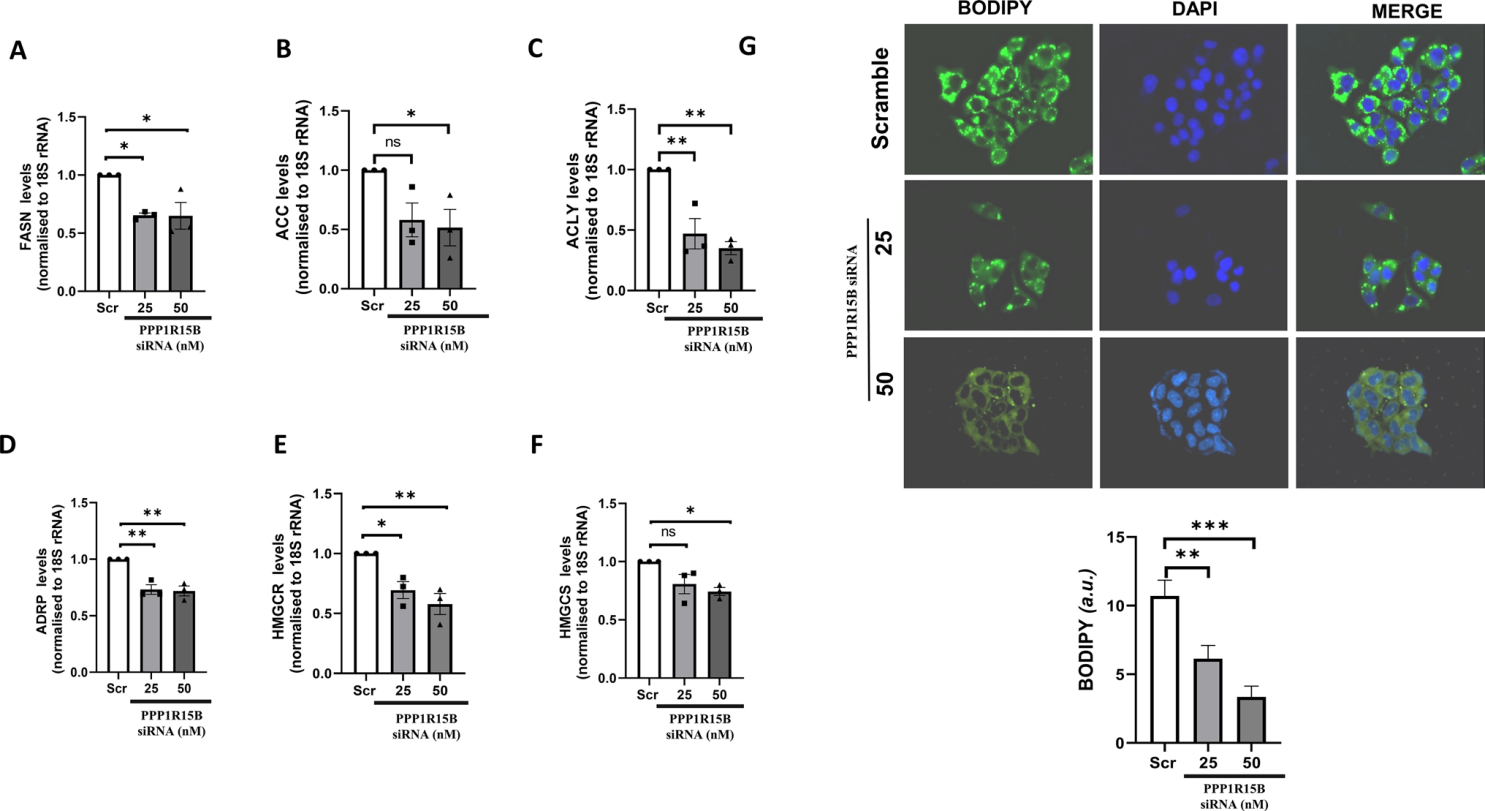
PPP1R15B inhibition reduces lipogenesis and hepatic steatosis in HepG2 cells. HepG2 cells were transfected with either the scramble (Scr) or PPP1R15B siRNA (25 and 50nM) and after 48 h, total RNA was isolated, reverse transcribed and subjected to qRT-PCR using primers for FASN **(A)**, ACC **(B)**, ACLY **(C)**, ADRP **(D)**, HMGCR **(E)** and HMGCS **(F)**. 18 S rRNA was used as the loading control. (E) HepG2 cells were grown on cover-slips placed on 24-well plates transfected as in “A”. After 48 h, cells were fixed with 4% formaldehyde and stained with Bodipy for 15 min. Nuclei were stained with DAPI. After thorough washing, cells were visualised in a fluorescent microscope. Quantification of the green fluorescence was done using the ImageJ software and is represented in the lower panel Experiments were performed in triplicate and values are presented as means ± SEM. ***P < 0.001, **P < 0.01 and *P < 0.05

Since PPP1R15B was validated as a target of miR-98-5p and this interaction was associated with altered lipid and glucose metabolism in HepG2 cells, we attempted to explore if PPP1R15B inhibition alone was sufficient to exert similar effects as miR-98-5p over-expression. HepG2 cells transfected with the scramble or PPP1R15B siRNA at 25 and 50nM dose for 48 h and this led to significant inhibition of PPP1R15B at the transcript and protein level (Fig. 5A, B). Similar to miR-98-5p effects, this was accompanied by a dose dependent increase in the levels of p-eIF2α in the presence of the siRNA without any change in the total levels of eIF2α (Fig. 5C). Interestingly, PPP1R15B inhibition alone was enough to significantly down-regulate the transcript levels of gluconeogenic genes (Fig. 5D-F) at a dose of 50 nM; however, FBP transcript levels remained unchanged (Fig. 5G). Also, hepatic glucose output decreased significantly in the presence of PPP1R15B siRNA (Fig. 5H). Moreover, mRNA levels of lipogenic genes such as FASN, ACC, ACLY, ADRP, HMGCR, HMGCS (Fig. 6A-F) and lipid accumulation (Fig. 6G) were significantly decreased upon PPP1R15B inhibition at 48 h post transfection.

### p-eIF2α mediated effects of miR-98-5p-PPP1R15B interaction on gluconeogenic and lipogenic gene expression is possibly through CREB

**Fig. 7:**
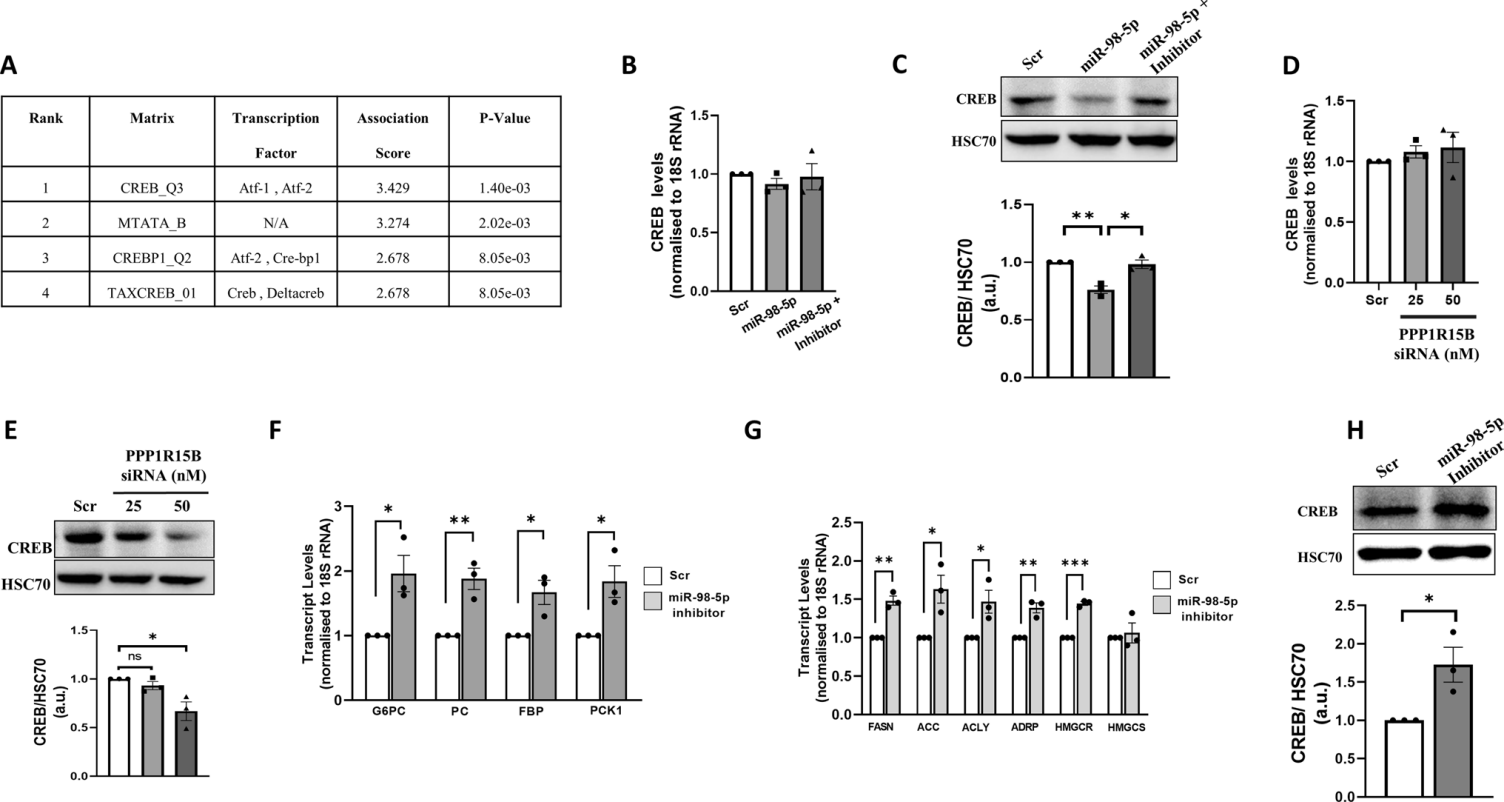
CREB mediates deregulated Gluconeogenesis and Lipogenesis in HepG2 cells. **(A)** Using the online tool, PASTAA, CREB emerged as the most over-represented transcription factor common to both gluconeogenic and lipogenic genes. **(B)** Cells were transfected with either the scramble or miR-98-5p mimic and/or inhibitor (75nM) and after 48 h, total RNA was isolated, reverse transcribed and subjected to qRT-PCR using primers for CREB. 18 S rRNA was used as the loading control. **(C)** Cells were transfected as in “A” and lysates (50 µg) were subjected to Western Blot analyses to detect the levels of CREB. HSC70 was used as the loading control. HepG2 cells were transfected with either the scramble (Scr) or PPP1R15B siRNA (25 and 50nM) and after 48 h, total RNA was isolated from cells and subjected to qRT-PCR to detect the transcript **(D)** and protein **(E)** levels of CREB. Cells were transfected with either the scramble or miR-98-5p inhibitor (75nM) and after 48 h, transcript levels of gluconeogenic (**F**: G6PC, PC, FBP and PCK1) and lipogenic (**G**: FASN, ACC, ACLY, ADRP and HMGCR) genes were evaluated by qRT-PCR. **(H)** HepG2 cells transfected as in “F” were lysed and the levels of CREB were detected by Western Blot analysis as in “E”. 18 S rRNA and HSC70 were used as the loading controls. Experiments were performed in triplicate and values are presented as means ± SEM. ***P < 0.001, **P < 0.01 and *P < 0.05

Deregulated hepatic gluconeogenesis and lipogenesis is almost always due to altered expression of gluconeogenic and lipogenic genes which is mediated by a complex interplay of transcription factors (TFs) and other regulators. To investigate the presence of such transcription factors we explored the enrichment of transcription factors on the altered genes using PASTAA tool that utilizes the prediction of binding affinities of a TF to a set of gene promoters. Using a cut-off p-value score of > 0.01, CREB_Q3 was found to be the most over-represented (Fig. 7A). Towards validation, HepG2 cells were transfected either with the scramble or with miR-98-5p mimic alone or together with its inhibitor for 48 h and upon completion of incubation the transcript and protein levels of CREB were assessed by qRT-PCR and Western Blot, respectively. While the transcript levels of CREB showed no change as shown in Fig. 7B, there was a significant decrease in protein levels in the presence of mimic miR-98-5p that was rescued in the presence of miR-98-5p inhibitor (Fig. 7C) suggesting that CREB might be an upstream regulator of gluconeogenesis and lipogenesis that is governed by altered levels of p-eIF2α in the presence of miR-98-5p. Also, inhibition of PPP1R15B using a specific siRNA decreased the protein levels of CREB without affecting its transcript levels (Fig. 7D, E).

#### mir-98-5p inhibition alone increases gluconeogenic and lipogenic genes’ transcription

Circulatory levels of miR-98-5p are decreased during diabetes(Khan et al. 2020) and to decipher a physiological relevance within the liver, we mimicked this state in HepG2 cells by inhibiting the miRNA levels using a specific inhibitor. As shown in Fig. 7F-G, miR-98-5p inhibition significantly increased the transcript levels of gluconeogenic (G6PC, PC, PCK and FBP) and lipogenic (FASN, ACC, ACLY, ADRP and HMGCR) genes. Also, corroborating with Fig. 7E, as compared to scramble, there was a significant increase in CREB protein levels in the presence of the miR-98-5p inhibitor (Fig. 7H).

**Fig. 8:**
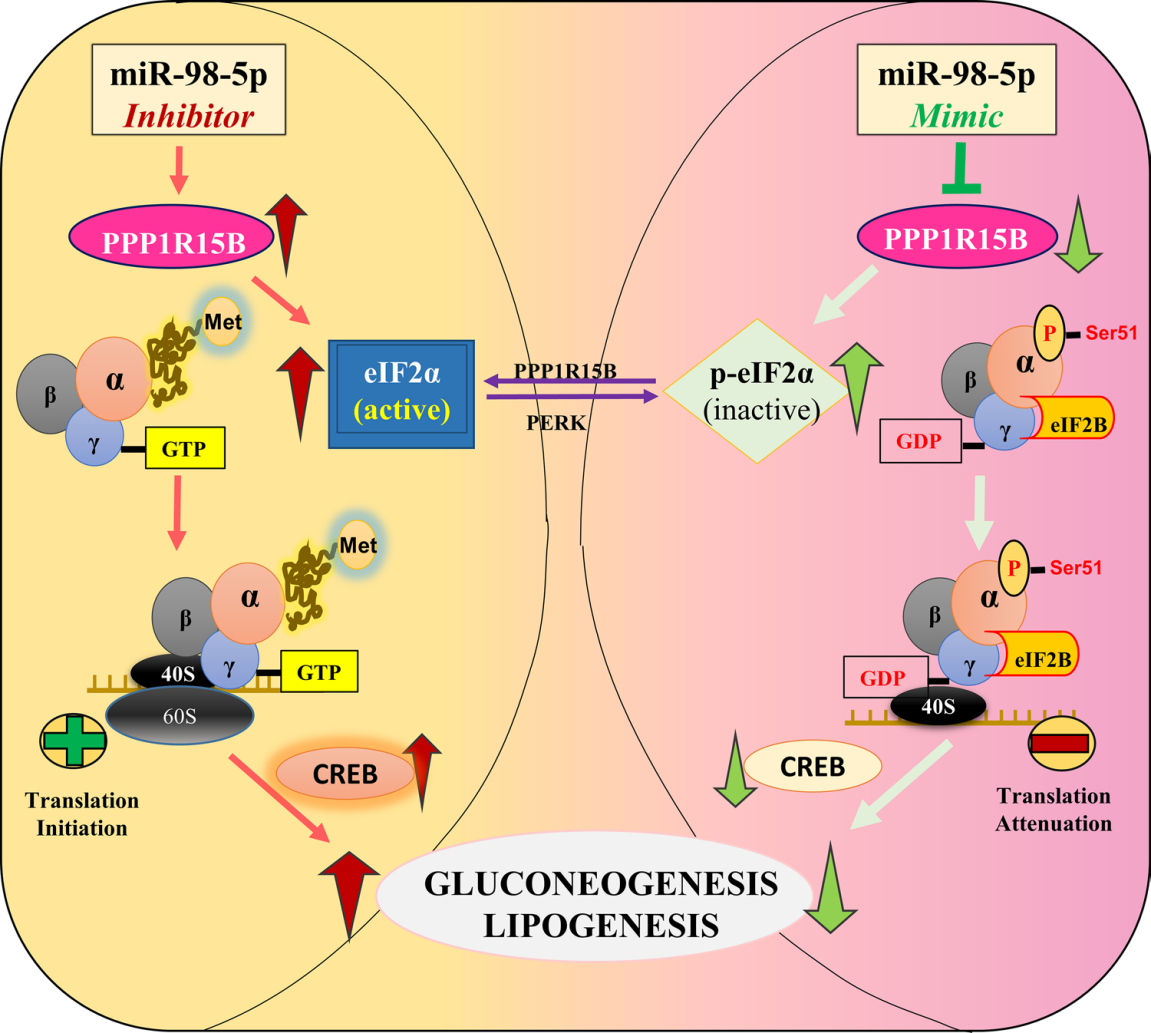
A schematic representation depicting the comparison of cellular consequences of mir-98-5p and its inhibitor. While our data using HepG2 cells in vitro demonstrate a reduction in gluconeogenesis and lipogenesis due to miR-98-5p overexpression; under *in-vivo* states where the circulatory levels of miR-98-5p are decreased, because PPP1R15B would not be targeted and inhibited, p-eIF2α levels would decrease with a concomitant increase in total eIF2α levels that possibly will increase gluconeogenic and lipogenic protein synthesis, consequently increasing hepatic glucose output and hepatic lipid accumulation, prominent phenotypes of diabetes

Collectively, our data show that miR-98-5p targets PPP1R15B and the miR-98-5p-PPP1R15B axis increases p-eIF2α levels which stalls CREB translation and the resulting decreased CREB protein levels facilitate a decrease in the levels of lipogenic and gluconeogenic genes. As opposed to this, when the levels of miR-98-5p are decreased as in the presence of the miRNA inhibitor, the levels of PPP1R15B are increased and this leads to increased levels of CREB, that consequently elevates gluconeogenic and lipogenic gene transcription (Fig. 8).

## Discussion

In this study we present evidence to show that miR-98-5p, whose levels are down-regulated in the circulation during diabetes, regulates hepatic gluconeogenesis and lipogenesis by targeting PPP1R15B. In the recent years, deregulated levels of circulatory miRNAs have been shown to induce aberrant gene expression patterns in metabolic diseases including diabetes (Mori et al. 2019; Pordzik et al. 2019). It is well established that circulatory miR’s are potential biomarkers in the diagnosis and prognosis of several diseases (Mori et al. 2019). Glucose intolerance and insulin resistance was induced in lean mice by injecting circulating extracellular vesicles (EVs) packed with miRNAs from obese mice (Ying et al. 2017, Castaño et al. 2018) and a similar effect was seen with synthetic EVs (Castaño et al. 2018); conversely when EVs from lean mice were induced into obese mice, there was an alleviation in insulin resistance (Ying et al. 2017). These indicate that miRNAs loaded onto EVs and present in the circulation have major roles in the regulation of metabolism within the cell and therefore harbor potential to serve as possible therapeutic agents.

A study from our laboratory demonstrated that circulatory levels of miR-98-5p, a member of miR let-7 family, are decreased in diabetic patients (Khan et al. 2020). Circulatory miR-98-5p levels are reported to be downregulated during diabetes and in other diseases like CAD(Pordzik et al. 2018; Sheikh 2021), diabetic nephropathy (Eroglu et al. 2020), neurological disorders(Yang et al. 2021). Also, miR-98 levels are downregulated in liver fibrotic models (Wang et al. 2020; Ma et al. 2022). The miR let-7 family codes for 9 mature miRNAs and collectively, they control stemness, metabolism, apoptosis, and cell proliferation. Several investigations have implicated let-7 in the control of glucose metabolism in a variety of tissues (Jiang 2019) and its target genes have been linked to T2D in human GWAS (Jiang 2019). Among a set of four clusters on the X-chromosome, miR-98 together with let-7f locates to the fourth cluster. In HCC patients, miR-98 acts as a tumor suppressor by regulating the Wnt/-catenin signaling through direct suppression of EZH2 expression (Zhang et al. 2017). By targeting IL-10, miR-98 prevents HCC from spreading in HCC-conditioned tumor-associated macrophages (TAMs) (Li et al. 2018) and by targeting NIK, miR-98-5p prevents HBV-HCC cells from proliferating, migrating, and invading (Fei et al. 2020). Our data shows that miR-98-5p targets PPP1R15B by binding to its 3’UTR and regulates its levels. PPP1R15B, a protein phosphatase that dephosphorylates eIF2α is associated with MODY (Abdulkarim et al. 2015, Kernohan et al. 2015) and two patients from a consanguineous family with homozygous missense mutation in PPP1R15B had juvenile onset diabetes, low height (Abdulkarim et al. 2015) and microcephaly, cerebral impairment, intellectual disability (Kernohan et al. 2015). Deficiency of PPP1R15B is associated with infantile cirrhosis, growth impairment, and neurodevelopmental anomalies(Mohammad et al. 2016).

However, little information is available regarding deregulated PPP1R15B expression and the relevance within the liver. A previous study from our laboratory demonstrated that in keratinocytes, miR-98-5p increased p-eIF2α levels by targeting PPP1R15B (Khan et al. 2020). Knockout studies reveal that PPP1R15B plays a prominent role in eIF2α dephosphorylation during mammalian development (Harding et al. 2009). eIF2α phosphorylation is a master regulator that controls selective translation of mRNAs necessary for adaptive functions of the cell and also represses global translation in response to various stresses. Mice with homozygous mutation of eIF2α (S51A) died immediately after birth due to accompanying hypoglycaemia as a result of defective gluconeogenesis (Scheuner et al. 2001). Conversely, adult mice that are heterozygous for the identical eIF2α S51A mutation are more likely to be obese, have insulin resistance, and glucose intolerance (Scheuner et al. 2005). Studies in PERK-null mice and in the human disease Wolcott-Rallison syndrome, a rare infantile-onset insulin-requiring diabetes caused by a loss-of-function mutation in the PERK gene, revealed the involvement of UPR and more specifically, the PERK-eIF2α pathway (Delépine et al. 2000, Zhang, Feng et al. 2006). These reports suggest that correlation between metabolic control and eIF2α phosphorylation is well established. Our data presented here demonstrates that miR-98-5p by targeting PPP1R15B promotes an accumulation of p-eIF2α in HepG2 cells which possibly decreases CREB protein levels that might be associated with aberrant hepatic metabolism. Using PASTAA, CREB emerged as the most enriched transcription factor and it is known to regulate PC, PCK1, and G6PC transcriptionally through recruitment of coactivators (Herzig et al. 2001, Oh, Han et al. 2013). CREB pathway has also been shown as an early signal in NAFLD progression in high-fat fed rats (Awaad et al. 2020). Moreover, fatty liver and increased hepatic TAG content were phenotypes observed in mice liver with dominant-negative CREB expression (Herzig et al. 2003). In fact, our data shows that there is decreased transcription of CREB-regulated gluconeogenic and lipogenic genes together with decreased glucose output and lipid accumulation in the presence of miR-98-5p; all suggestive of a crucial role of the miR-98-5p/PPP1R15B/eIF2α axis in deregulated hepatic metabolism as is evident in obesity, insulin resistance and diabetes. Since an earlier report from our laboratory had demonstrated decreased circulatory levels of miR-98-5p in diabetic subjects (Khan et al. 2020), we think that by modulating the PPP1R15B/eIF2α axis, decreased miR-98-5p levels might increase hepatic gluconeogenesis and lipogenesis, two evident hallmarks of aberrant hepatic physiology. This was apparent in the presence of miR-98-5p inhibitor alone where there was increased transcription of gluconeogenic and lipogenic genes in HepG2 cells. Increased hepatic gluconeogenesis, lipogenesis and glucose output are major contributors of the diabetic phenotype and these are elevated in diabetic animal models and diabetic subjects (Dorn et al. 2010, Eissing, Scherer et al. 2013, Zhang, Xu et al. 2013).

To conclude, our data identifies the miR-98-5p/PPP1R15B axis as a significant regulator of hepatic gluconeogenesis and lipogenesis and offers a possible path to explore the therapeutic potential of this axis to target deregulated hepatic metabolism during metabolic diseases.

## Data Availability

All the available data are present in the manuscript.

## Abbreviations

miRNAs: MicroRNAs
PPP1R15B: Protein phosphatase 1 regulatory subunit 15B
p-eIF2α: Eukaryotic translation initiation factor 2a
NAFLD: Nonalcoholic fatty liver disease
T2DM: Type 2 diabetic mellitus
CREB: cAMP response element-binding protein
EVs: Extracellular vesicles
qRT-PCR: Quantitative real time PCR
PCK1: Phosphoenolpyruvate carboxykinase
G6PC: Glucose 6 phosphatase
FBP: Fructose 1,6-bisphosphatase
PC: Pyruvate carboxylase
ADRP: Adipose differentiation-related protein
ACC: Acetyl CoA carboxylase
ACLY: ATP citrate lyase
FASN: Fatty acid synthase
HMGCR: HydroxyMethyl glutaryl-CoA reductase
HMGCS: HydroxyMethyl glutaryl-CoA synthase
CREB: cAMP-responsive element-binding protein
HCC: Hepatocellular carcinoma
DNL: de novo lipogenesis
NIK: Nuclear factor-κb-Inducing kinase
EZH2: Enhancer of zeste homolog 2
TAMs: HCC-conditioned tumor-associated macrophages
CAD: Coronary artery disease
HBV-HCC: HepatitisB virus-related hepatocellular carcinoma

## References

[CR1] Abdulkarim B, Nicolino M, Igoillo-Esteve M, Daures M, Romero S, Philippi A, Senée V, Lopes M, Cunha DA, Harding HP (2015). A missense mutation in PPP1R15B causes a syndrome including diabetes, short stature and microcephaly. Diabetes.

[CR2] Awaad AK, Kamel MA, Mohamed MM, Helmy MH, Youssef MI, Zaki EI, Essawy MM, Hegazy MG (2020). The role of hepatic transcription factor cAMP response element-binding protein (CREB) during the development of experimental nonalcoholic fatty liver: a biochemical and histomorphometric study. Egypt Liver J.

[CR3] Castaño C, Kalko S, Novials A, Párrizas M (2018). Obesity-associated exosomal miRNAs modulate glucose and lipid metabolism in mice. Proc Nat Acad Sci.

[CR4] Delépine M, Nicolino M, Barrett T, Golamaully M, Mark Lathrop G, Julier C (2000). EIF2AK3, encoding translation initiation factor 2-α kinase 3, is mutated in patients with Wolcott-Rallison syndrome. Nat Genet.

[CR5] Dorn C, Riener M-O, Kirovski G, Saugspier M, Steib K, Weiss TS, Gäbele E, Kristiansen G, Hartmann A, Hellerbrand C (2010). Expression of fatty acid synthase in nonalcoholic fatty liver disease. Int J Clin Exp Pathol.

[CR6] Eissing L, Scherer T, Tödter K, Knippschild U, Greve JW, Buurman WA, Pinnschmidt HO, Rensen SS, Wolf AM, Bartelt A (2013). De novo lipogenesis in human fat and liver is linked to ChREBP-β and metabolic health. Nat Commun.

[CR7] Eroglu İ, Korkmaz H, Ozturk KH, Sirin FB, Sevik S, Afsar B (2020). New risk factors in diabetic nephropathy: microRNA-196-3p and microRNA-203. Minerva Endocrinol.

[CR8] Fei X, Zhang P, Pan Y, Liu Y (2020). MicroRNA-98-5p inhibits tumorigenesis of hepatitis B virus-related hepatocellular carcinoma by targeting NF-κB-inducing kinase. Yonsei Med J.

[CR9] Gaggini M, Morelli M, Buzzigoli E, DeFronzo RA, Bugianesi E, Gastaldelli A (2013). Non-alcoholic fatty liver disease (NAFLD) and its connection with insulin resistance, dyslipidemia, atherosclerosis and coronary heart disease. Nutrients.

[CR10] Galicia-Garcia U, Benito-Vicente A, Jebari S, Larrea-Sebal A, Siddiqi H, Uribe KB, Ostolaza H, Martín C (2020). Pathophysiology of type 2 diabetes mellitus. Int J Mol Sci.

[CR11] Harding HP, Zhang Y, Scheuner D, Chen J-J, Kaufman RJ, Ron D (2009). Ppp1r15 gene knockout reveals an essential role for translation initiation factor 2 alpha (eIF2α) dephosphorylation in mammalian development. Proc Nat Acad Sci.

[CR12] Herzig S, Long F, Jhala US, Hedrick S, Quinn R, Bauer A, Rudolph D, Schutz G, Yoon C, Puigserver P (2001). CREB regulates hepatic gluconeogenesis through the coactivator PGC-1. Nature.

[CR13] Herzig S, Hedrick S, Morantte I, Koo S-H, Galimi F, Montminy M (2003). CREB controls hepatic lipid metabolism through nuclear hormone receptor PPAR-γ. Nature.

[CR14] Huang B, Huang L-F, Zhao L, Zeng Z, Wang X, Cao D, Yang L, Ye Z, Chen X, Liu B (2020). Microvesicles (MIVs) secreted from adipose-derived stem cells (ADSCs) contain multiple microRNAs and promote the migration and invasion of endothelial cells. Genes&nbsp; Dis.

[CR15] Hurtado MD, Vella A (2019). What is type 2 diabetes?. Medicine.

[CR16] Jansen F, Yang X, Hoelscher M, Cattelan A, Schmitz T, Proebsting S, Wenzel D, Vosen S, Franklin BS, Fleischmann BK (2013). Endothelial microparticle–mediated transfer of microRNA-126 promotes vascular endothelial cell repair via SPRED1 and is abrogated in glucose-damaged endothelial microparticles. Circulation.

[CR17] Jiang S (2019). A regulator of metabolic reprogramming: MicroRNA Let-7. Translational oncology.

[CR18] Kamalden TA, Macgregor-Das AM, Kannan SM, Dunkerly-Eyring B, Khaliddin N, Xu Z, Fusco AP, Yazib SA, Chow RC, Duh EJ (2017). Exosomal microRNA-15a transfer from the pancreas augments diabetic complications by inducing oxidative stress. Antioxid Redox Signal.

[CR19] Katayama M, Wiklander OP, Fritz T, Caidahl K, El-Andaloussi S, Zierath JR, Krook A (2019). Circulating exosomal miR-20b-5p is elevated in type 2 diabetes and could impair insulin action in human skeletal muscle. Diabetes.

[CR20] Kernohan KD, Tétreault M, Liwak-Muir U, Geraghty MT, Qin W, Venkateswaran S, Davila J, Consortium CRC, Holcik M, Majewski J (2015). Homozygous mutation in the eukaryotic translation initiation factor 2alpha phosphatase gene, PPP1R15B, is associated with severe microcephaly, short stature and intellectual disability. Hum Mol Genet.

[CR21] Khan R, Kadamkode V, Kesharwani D, Purkayastha S, Banerjee G, Datta M (2020). Circulatory mir-98-5p levels are deregulated during diabetes and it inhibits proliferation and promotes apoptosis by targeting PPP1R15B in keratinocytes. RNA Biol.

[CR22] Kitade H, Chen G, Ni Y, Ota T (2017). Nonalcoholic fatty liver disease and insulin resistance: new insights and potential new treatments. Nutrients.

[CR23] Li L, Sun P, Zhang C, Li Z, Zhou W (2018). MiR-98 suppresses the effects of tumor-associated macrophages on promoting migration and invasion of hepatocellular carcinoma cells by regulating IL-10. Biochimie.

[CR24] Loria P, Lonardo A, Anania F (2013). Liver and diabetes. A vicious circle. Hepatol Res.

[CR25] Ludwig N, Leidinger P, Becker K, Backes C, Fehlmann T, Pallasch C, Rheinheimer S, Meder B, Stähler C, Meese E (2016). Distribution of miRNA expression across human tissues. Nucleic Acids Res.

[CR26] Ma Y, Yuan X, Han M, Xu Y, Han K, Liang P, Liu S, Chen J, Xing H (2022). miR-98-5p as a novel biomarker suppress liver fibrosis by targeting TGFβ receptor 1. Hepatol Int.

[CR27] Mohammad S, Wolfe LA, Stöbe P, Biskup S, Wainwright MS, Melin-Aldana H, Malladi P, Muenke M, Gahl WA, Whitington PF (2016). Infantile cirrhosis, growth impairment, and neurodevelopmental anomalies associated with deficiency of PPP1R15B. J Pediatr.

[CR28] Mori MA, Ludwig RG, Garcia-Martin R, Brandão BB, Kahn CR (2019). Extracellular miRNAs: from biomarkers to mediators of physiology and disease. Cell Metabol.

[CR29] Oh K-J, Han H-S, Kim M-J, Koo S-H (2013). CREB and FoxO1: two transcription factors for the regulation of hepatic gluconeogenesis. BMB Rep.

[CR30] Ota T, Takamura T, Kurita S, Matsuzawa N, Kita Y, Uno M, Akahori H, Misu H, Sakurai M, Zen Y (2007). Insulin resistance accelerates a dietary rat model of nonalcoholic steatohepatitis. Gastroenterology.

[CR31] Pordzik J, Pisarz K, De Rosa S, Jones AD, Eyileten C, Indolfi C, Malek L, Postula M (2018). The potential role of platelet-related microRNAs in the development of cardiovascular events in high-risk populations, including diabetic patients: a review. Front Endocrinol.

[CR32] Pordzik J, Jakubik D, Jarosz-Popek J, Wicik Z, Eyileten C, De Rosa S, Indolfi C, Siller-Matula JM, Czajka P, Postula M (2019). Significance of circulating microRNAs in diabetes mellitus type 2 and platelet reactivity: bioinformatic analysis and review. Cardiovasc Diabetol.

[CR33] Roider HG, Manke T, O’Keeffe S, Vingron M, Haas SA (2009). PASTAA: identifying transcription factors associated with sets of co-regulated genes. Bioinformatics.

[CR34] Rui L (2014). Energy metabolism in the liver. Compr Physiol.

[CR35] Sapp RM, Hagberg JM (2019). Circulating microRNAs: advances in exercise physiology. Curr Opin Physiol.

[CR36] Scheuner D, Song B, McEwen E, Liu C, Laybutt R, Gillespie P, Saunders T, Bonner-Weir S, Kaufman RJ (2001). Translational control is required for the unfolded protein response and in vivo glucose homeostasis. Mol Cell.

[CR37] Scheuner D, Mierde DV, Song B, Flamez D, Creemers JW, Tsukamoto K, Ribick M, Schuit FC, Kaufman RJ (2005). Control of mRNA translation preserves endoplasmic reticulum function in beta cells and maintains glucose homeostasis. Nat Med.

[CR38] Sheikh MSA (2021). Role of plasma soluble lectin-like oxidized low-density lipoprotein receptor-1 and microRNA-98 in severity and risk of coronary artery disease. Balkan Med J.

[CR39] Valadi H, Ekström K, Bossios A, Sjöstrand M, Lee JJ, Lötvall JO (2007). Exosome-mediated transfer of mRNAs and microRNAs is a novel mechanism of genetic exchange between cells. Nat Cell Biol.

[CR40] Wang Q, Wei S, Zhou H, Li L, Zhou S, Shi C, Shi Y, Qiu J, Lu L (2020). MicroRNA-98 inhibits hepatic stellate cell activation and attenuates liver fibrosis by regulating HLF expression. Front cell Dev Biol.

[CR41] Xu Q, Li Y, Shang Y-F, Wang H-L, Yao M-X (2015). miRNA-103: molecular link between insulin resistance and nonalcoholic fatty liver disease. World J Gastroenterol.

[CR42] Yáñez-Mó M, Siljander PR-M, Andreu Z, Bedina Zavec A, Borràs FE, Buzas EI, Buzas K, Casal E, Cappello F, Carvalho J (2015). Biological properties of extracellular vesicles and their physiological functions. J Extracell Vesicles.

[CR43] Yang J, Cao L-L, Wang X-P, Guo W, Guo R-B, Sun Y-Q, Xue T-F, Cai Z-Y, Ji J, Cheng H (2021). Neuronal extracellular vesicle derived miR-98 prevents salvageable neurons from microglial phagocytosis in acute ischemic stroke. Cell Death Dis.

[CR44] Ying W, Riopel M, Bandyopadhyay G, Dong Y, Birmingham A, Seo JB, Ofrecio JM, Wollam J, Hernandez-Carretero A, Fu W (2017). Adipose tissue macrophage-derived exosomal miRNAs can modulate in vivo and in vitro insulin sensitivity. Cell.

[CR45] Zhang W, Feng D, Li Y, Iida K, McGrath B, Cavener DR (2006). PERK EIF2AK3 control of pancreatic β cell differentiation and proliferation is required for postnatal glucose homeostasis. Cell Metabol.

[CR46] Zhang F, Xu X, Zhang Y, Zhou B, He Z, Zhai Q (2013). Gene expression profile analysis of type 2 diabetic mouse liver. PLoS ONE.

[CR47] Zhang J-J, Chen J-T, Hua L, Yao K-H, Wang C-Y (2017). miR-98 inhibits hepatocellular carcinoma cell proliferation via targeting EZH2 and suppressing Wnt/β-catenin signaling pathway. Biomed Pharmacother.

